# Unilateral renal fungus ball caused by *Candida glabrata*

**DOI:** 10.1016/j.mmcr.2024.100649

**Published:** 2024-04-14

**Authors:** Katrina J. Villegas, Nagihan Orhun, Sebastian Vera Garces, Sacide S. Ozgur, Patrick Michael, Ali Zahran, Daniel Rabinowitz

**Affiliations:** a St. Joseph's University Medical Center, Department of Internal Medicine, 703 Main St, Paterson, NJ, 07503, USA; b St. George's University School of Medicine, Grenada; c St. Joseph's University Medical Center, Department of Infectious Disease, 703 Main St, Paterson, NJ, 07503, USA; d St. Joseph's University Medical Center, Department of Urology, 703 Main St, Paterson, NJ, 07503, USA

**Keywords:** Fungus ball, Candida, Nephrolithiasis, Urinary tract infection

## Abstract

A 46-year-old diabetic woman with unilateral renal fungus ball was successfully treated with antifungal therapy, endoscopic extraction and ureteral stent placement. The patient was initially treated for a right staghorn calculus, thereafter developed urinary symptoms. Imaging revealed distal left ureter filling defects and a previous stent at the ureteropelvic junction. Urine culture confirmed *Candida glabrata* sensitive to Micafungin. Bilateral ureteroscopy facilitated the extraction of a left renal pelvis fungus ball. This case underscores the challenges in diagnosing fungal UTIs in patients with predisposing factors, and highlights a combined medical and surgical approach for effective treatment of renal fungus balls.

## 1 Introduction

Renal fungus ball or bezoar is a collection of mycelial cells and sloughed off epithelial lining of the urinary tract. First described by Chrisholm in 1961 [1], this is usually seen in immunosuppressed patients, including diabetics and chronic alcoholics, and those who underwent prolonged catheterization or had prolonged antibiotic use. It is a rare complication of a fungal urinary tract infection, mostly due to *Candida* species [2], that may eventually lead to fungemia, obstructive uropathy, renal failure and bladder rupture. Management is both medical and surgical, warranting prompt recognition and treatment to avoid further complications [[2], [3], [4]]. Schonebeck and Ansehn noted only 29 cases of upper renal tract fungal bezoars. Despite increasing number of cases over the years, fungus balls are still rarely encountered in clinical practice, and therefore, a robust evidence-based standard treatment has not been concluded [5].

We herein present a case of infected renal stones that has failed initial treatment with several courses of antibiotics, then was later found to have unilateral renal fungus ball secondary to *Candida glabrata* that was successfully treated by endoscopic extraction of the fungus ball and ureteral stent placement followed by retrograde irrigation of the bladder with Amphotericin B and intravenous Micafungin.

## 2 Case presentation

**Fig. 1:**
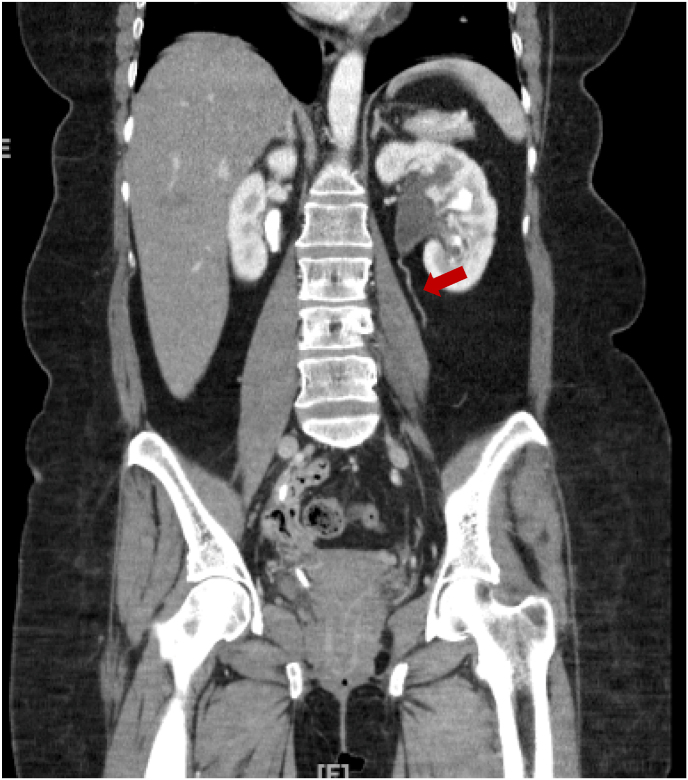
Coronal view of CT Abdomen/Pelvis with contrast taken a month prior. *Red arrow* points to small filling defects in the distal left ureter, that was due to fungus balls found intraoperatively. (For interpretation of the references to colour in this figure legend, the reader is referred to the Web version of this article.)

A 46-year-old woman with a history of diabetes and nephrolithiasis presented on day 0 at the emergency room with suprapubic pain for the past 2 weeks. At day −30, the patient underwent percutaneous nephrolithotomy, stent placement, flexible nephroscopy and anterograde ureteroscopy due to a right staghorn calculus. The patient suffered from recurrent urinary tract infections thereafter. At day −7, the patient presented to the emergency room with complaints of dysuria. Computed Tomography Abdomen/Pelvis (CTAP) without contrast was performed, and revealed that a previous staghorn calculus has been removed, with a right nephro-ureteral stent at the level of the UPJ present. It also showed small filling defects seen in the distal left ureter, that had been noted previously about a month prior when she had a CTAP with contrast prior to undergoing the urologic procedures (Fig. 1).

On day 0, the pain did not radiate but was associated with dysuria and dark urine. The patient also noted episodes of chills at home with no fever. Home medications included Sitagliptin-Metformin 50/500mg 2 tablets orally daily and Insulin Glargine 20 units subcutaneously every night. Upon presentation, the patient's vital signs were stable with a blood pressure of 132/84 mmHg, heart rate of 97 per minute, respiratory rate of 16 per minute, 99 % oxygen saturation and a temperature of 37.1 Celsius. IV antibiotics were started due to the patient's history and a positive urine dipstick. The patient was admitted for persistent urinary symptoms and persistent suprapubic pain despite outpatient treatment. She was scheduled for removal of a right sided stent and would be monitored leading up to the procedure.

During the hospital course, a preliminary urine culture result grew yeast. Intravenous antibiotics were discontinued, and empiric Fluconazole 400mg IV every 24 hours was started. Patient underwent bilateral ureteroscopy with left ureteral stent insertion. During the procedure, a fungus ball was found in the left renal pelvis and some of the calyces. Extraction of the fungus ball was done. Due to the amount of fungus ball and inflammatory material that was removed, it was determined that a left ureteral stent would be placed to allow for any residual debris that was not clearly visualized during surgery to drain out and that a Foley catheter be placed. The patient tolerated the surgery well. The final urine culture showed *Candida glabrata*, for which Fluconazole was discontinued. Micafungin 100mg IV every 24 hours was then given, as well as Amphotericin B 50mg diluted in 1000ml distilled water through the Foley catheter every 24 hours as a flush to the urinary bladder wall. The urine specimen was sent for antifungal sensitivity specific to Micafungin, which showed that it was sensitive, with a Minimal Inhibitory Concentration (MIC) dilution of ≤0.06. The removed fungus ball was not subjected to microbiological or histopathological investigation, although a repeat urine culture from the left renal pelvis collected during the surgery confirmed the same inciting organism, *C. glabrata*. She received 2 days of Amphotericin B urinary bladder flushes, after which the left ureteral stent and Foley catheter were removed. She was discharged home following a 9-day hospital stay which included surgery, and 7 days of antifungal treatment with IV Micafungin. Following her discharge, the patient received an additional 7-day outpatient course of Micafungin 100mg IV every 24 hours through midline access, completing a 14-day course. She was able to successfully tolerate the outpatient therapy. She had no adverse effects during the treatment period. During her follow-up clinic visits with Infectious Disease and Urology, our patient no longer complained of dysuria, chills or suprapubic pain. Subsequent urinalysis showed no recurrence of fungus in the urine.

## 3 Discussion

Urinary tract or renal fungus balls are formed when a necrotic tissue nucleus, mucous debris, and foreign or lithiasic debris concentrate. These are relatively uncommon and are considered severe complications of urinary fungal infections [6]. Two well-known causes of urinary tract fungus balls are ascending candiduria and hematogenous dissemination of candidiasis, in 85% of which renal parenchymal involvement and high mortality rate are observed [7].

The prevailing pathogens associated with fungal urinary tract infections are typically *Candida albicans* and *Candida tropicalis* [8]. *Candida glabrata* constitutes approximately 20% of *Candida* urinary tract infections, and despite its lack of hyphae production, it is still capable of causing renal abscesses and forming fungal balls [9]. Predisposing factors for urinary *Candida* infections encompass diabetes mellitus, prolonged utilization of Foley catheters, urinary tract abnormalities, extended courses of antibiotic therapy, immunosuppressive treatment, malnutrition, and malignancy [10]. As with our patient, renal fungus ball may be due to an ascending infection after a urologic procedure, as well as previous antibiotic treatment [9,10].

Timely diagnosis and early initiation of appropriate treatment of urinary tract fungus balls are essential due to the high risk of obstructive uropathy and fungal urosepsis, particularly in those with complex fungal infections, meaning that the upper urinary tract is affected [11]. However, diagnosis of fungal bezoars remains challenging for clinicians for several reasons. Because of the similarity of the clinical presentation (i.e. hematuria, costovertebral angle tenderness, dysuria) to the common pathologies affecting the urinary tract, including nephrolithiasis and pyelonephritis, as well as their rare occurrence, fungus balls might not be top-ranked in the differentials [12]. On imaging, renal fungal bezoars are commonly visualized as filling defects in the pelvicalyceal system in intravenous pyelography, and as obstructing echogenic masses in CT scans and sonography [13]. These radiological proofs lack specificity as other urinary tract pathologies, such as blood clots, polyps, urinary calculi, and epithelial tumors, can also present with similar imaging findings [14]. Therefore, maintaining a high level of clinical suspicion by combining imaging findings with a positive fungal urine culture, particularly in patients with predisposing conditions, may enable definitive diagnosis.

The previous literature, mostly case reports, showed that a combination of medical and interventional approaches was necessitated, whereas only medical management was the preferred approach in poor surgical candidates [5,14]. For *C. glabrata* UTIs with fungus balls, surgical intervention, systemic antifungal treatment based on susceptibility with either Fluconazole, Amphotericin B or Flucytosine, and irrigation through a nephrostomy tube with Amphotericin B are recommended [15]. Echinocandins generally do not reach the urinary tract, and are not preferred to treat *Candida* UTIs. However, Micafungin has been reported to successfully treat persistent UTIs caused by *C. glabrata*, and is therefore an effective and safe alternative for its treatment [16]. The most frequently documented therapeutic strategy entails urinary drainage utilizing a nephrostomy or a ureteral catheter accompanied by prolonged systemic and localized administration of antifungal agents, as was done with our patient [14,15]. We consider our case a valuable addition to the growing literature regarding the diagnosis and effective treatment of urinary tract fungus balls.

Renal fungus ball is rarely encountered in clinical practice. This case describes a *Candida glabrata* fungus ball that was successfully treated with endoscopic extraction and ureteral stent placement, followed by retrograde irrigation with Amphotericin B and systemic Micafungin therapy. In a case where a urinary tract infection has failed multiple treatments with antibiotics in a diabetic patient, clinicians should further investigate a possible fungal cause. Early recognition and aggressive treatment are essential to prevent complications that lead to a worse prognosis.

## Declaration of competing interest

The authors report no conflict of interest. An ethical review is not necessary because this is a case report. This research received no specific grant from funding agencies in the public, commercial, or not-for-profit sectors.

## CRediT authorship contribution statement

**Katrina J. Villegas:** Writing – review & editing, Writing – original draft, Conceptualization. **Nagihan Orhun:** Writing – original draft. **Sebastian Vera Garces:** Writing – original draft. **Sacide S. Ozgur:** Validation. **Patrick Michael:** Supervision. **Ali Zahran:** Supervision. **Daniel Rabinowitz:** Supervision.

## References

[1] Chisholm E.R., Hutch J.A. (1961). Fungus ball (Candida albicans) formation in the bladder. J. Urol..

[2] Kauffman C.A. (2014). Diagnosis and management of fungal urinary tract infection. Infect. Dis. Clin..

[3] Comiter C.V., McDonald M., Minton J. (1996). Fungal bezoar and bladder rupture secondary to Candida tropicalis. Urology.

[4] Shimada S., Nakagawa H., Shintaku I. (2006). Acute renal failure as a result of bilateral ureteral obstruction by Candida albicans fungus balls. Int. J. Urol..

[5] Rohloff M., Shakuri-Rad J., DeHaan A.P. (2017 Dec 1). Candida bezoars in Adults: determining optimal management. J Endourol Case Rep.

[6] Jegannathan D., Ramanathan K. (2016 Aug 1). Renal fungal ball—two case reports and review of literature. BJR Case Rep.

[7] Irby P.B., Stoller M.L., McAninch J.W. (1990 Mar 1). Fungal bezoars of the upper urinary tract. J. Urol..

[8] Praz V., Burruni R., Meid F., Wisard M., Jichlinski P., Tawadros T. (2014). Fungal ball in urinary tract, a rare entity, which needs a specific approach. Can Urol Assoc J..

[9] Fisher J.F., Kavanagh K., Sobel J.D., Kauffman C.A., Newman C.A. (2011). Candida urinary tract infection: pathogenesis. Clin. Infect. Dis..

[10] Abuelnaga M., Khoshzaban S., Badr M.R., Chaudry A. (2019 Dec 24). Successful endoscopic management of a renal fungal ball using flexible ureterorenoscopy. Case Rep Urol.

[11] Wainstein M.A., Graham R.C., Resnick M.I. (1995 Jul 1). Predisposing factors of systemic fungal infections of the genitourinary tract. J. Urol..

[12] Das M.K., Pakshi R.R., Kalra S., Elumalai A., Theckumparampil N. (2019 Dec 1). Fungal balls mimicking renal calculi: a zebra among horses. J Endourol Case Rep.

[13] Kale H., Narlawar R.S., Rathod K. (2002). Renal fungal ball: an unusual sonographic finding. J. Clin. Ultrasound.

[14] Zeineddine N., Mansour W., Bitar S.E., Campitelli M., Mobarakai N. (2017 Jan 1;2017). Fungal bezoar: a rare cause of ureteral obstruction. Case Rep Infect Dis.

[15] Pappas P.G., Kauffman C.A., Andes D.R., Clancy C.J., Marr K.A., Ostrosky-Zeichner L. (2016). Clinical practice guideline for the management of candidiasis: 2016 update by the infectious diseases society of America. Clin. Infect. Dis.: an official publication of the Infectious Diseases Society of America [Internet].

[16] Lagrotteria D., Rotstein C., Lee C.H. (2007 Jan 1). Treatment of candiduria with Micafungin: a case series. Can. J. Infect Dis. Med. Microbiol..

